# Switching hemodialysis patients from sevelamer hydrochloride to bixalomer: a single-center, non-randomized analysis of efficacy and effects on gastrointestinal symptoms and metabolic acidosis

**DOI:** 10.1186/1471-2369-14-222

**Published:** 2013-10-12

**Authors:** Shingo Hatakeyama, Hiromi Murasawa, Takuma Narita, Masaaki Oikawa, Naoki Fujita, Hiromichi Iwamura, Joutaro Mikami, Yuta Kojima, Tendo Sato, Ken Fukushi, Yusuke Ishibashi, Yasuhiro Hashimoto, Takuya Koie, Hisao Saitoh, Tomihisa Funyu, Chikara Ohyama

**Affiliations:** 1 Department of Urology, Graduate School of Medicine, Hirosaki University, 5 Zaifu-chou, 036-8562 Hirosaki, Japan; 2 Department of Urology, Oyokyo Kidney Research Institute, 036-8243 Hirosaki, Japan; 3 Department of Advanced Transplant and Regenerative Medicine, Graduate School of Medicine, Hirosaki University, 036-8562 Hirosaki, Japan

**Keywords:** Bixalomer, Hyperphosphatemia, Hemodialysis, Gastrointestinal disorder, Metabolic acidosis

## Abstract

**Background:**

Bixalomer (BXL) was developed to improve gastrointestinal symptoms and reduce constipation, relative to sevelamer hydrochloride, in hemodialysis patients. We prospectively evaluated the safety and effectiveness of switching maintenance dialysis patients from sevelamer hydrochloride to BXL.

**Methods:**

Twenty-eight patients were switched from sevelamer hydrochloride to BXL (1:1 dose) from July to October 2012, whereas 84 randomly selected patients not treated with sevelamer hydrochloride were enrolled as a control group. The primary endpoint was improvement of gastrointestinal symptoms; secondary endpoints included improvement in metabolic acidosis, changes in blood biochemistry, and safety 12 weeks after the switch. We also surveyed patient satisfaction with switching to BXL 12 weeks after the switch.

**Results:**

Before switching, symptoms of epigastric fullness were significantly worse in the switch than in the control group. Twelve weeks after the switch, reflux, epigastric fullness, and constipation had improved significantly in the switch group. Other factors, including stomach ache, diarrhea, and form of stool, did not change significantly. Blood gas analysis showed that metabolic acidosis was significantly improved by switching. Four patients (14%) experienced grade 1 adverse events, all of which improved immediately after stopping BXL. Major adverse events were diarrhea and abdominal discomfort. Mean satisfaction score was 3.1 ± 0.7, with 64% of patients reporting they were “neither satisfied nor dissatisfied” after switching.

**Conclusions:**

A switch from sevelamer hydrochloride to BXL improved symptoms of reflux, epigastric fullness, constipation, and metabolic acidosis in hemodialysis patients.

**Trial registration:**

The study was registered as Clinical trial: (UMIN000011150).

## Background

Hyperphosphatemia is highly prevalent in patients undergoing hemodialysis and is one of the most important risk factors for cardiovascular disease and mortality in these patients [1]. A high serum level of phosphorous promotes calcium phosphate calcifications. Calcification of the myocardium or peripheral artery significantly increases the risk of cardiovascular disease in dialysis patients [2]. In most patients, dietary restrictions and three times-weekly hemodialysis sessions are insufficient to reduce phosphate levels to the treatment goals of the National Kidney Foundation (NKF K/DOQI) [3]. Thus, phosphate-binding agents are necessary for dialysis patients with hyperphosphatemia.

Currently available therapeutic agents for hyperphosphatemia include calcium-containing phosphate binders (calcium carbonate and calcium acetate), lanthanum carbonate, and ion-exchange resins. Sevelamer hydrochloride is an ion-exchange resin of non-absorbable hydrogel that reduces serum phosphorus levels. The disadvantages of this agent include gastrointestinal symptoms due to high pill burden [4] and metabolic acidosis [5]. Gastrointestinal adverse events occur frequently in Japanese dialysis patients, preventing their long-term treatment with sevelamer hydrochloride [6]. Metabolic acidosis not only has adverse effects on bone, and on protein and amino-acid metabolism, but reduces overall survival [7]. Metabolic acidosis caused by sevelamer hydrochloride may be reduced by the use of sevelamer carbonate, but the latter is unavailable in Japan.

Bixalomer (BXL) is a non-calcium, metal-free amine-functional polymer that reduces serum phosphorus levels by binding to phosphate in the gastrointestinal tract and inhibiting its absorption. A phase III trial of hemodialysis patients in Japan showed the clinical efficacy of BXL in reducing serum phosphorus levels, as well as its preferable long-term safety profile [8]. BXL was therefore approved in June 2012 to treat hyperphosphatemia in Japan. The lower rates of gastrointestinal symptoms and metabolic acidosis observed with BXL relative to sevelamer hydrochloride have been attributed to its lower coefficient of expansion and the absence of chloride [9].

We prospectively investigated the effectiveness and safety of a 1:1 dose switch from sevelamer hydrochloride to BXL in stable hemodialysis patients, including the effects of the switch on gastrointestinal symptoms, metabolic acidosis, and blood biochemistry, as well as patient satisfaction.

## Methods

### Study design

This was a single-center, open-label, non-randomized controlled study performed in hemodialysis patients with hyperphosphatemia. This study was performed in accordance with the ethical standards of the Declaration of Helsinki and was approved by the institutional ethics committee of the Oyokyo Kidney Research Institute, Hirosaki, Japan. All patients provided written informed consent.

**Figure 1:**
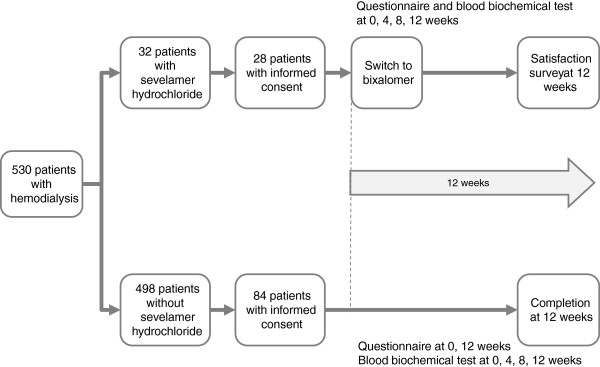
**Study design and disposition of subjects.** A total of 112 patients were enrolled in this study. Questionnaires were administered and blood biochemistry and blood gas were assessed at 0 (baseline), 4, 8, and 12 weeks after the switch in the switch group. In the control group, questionnaires were administered at 0 and 12 weeks, and blood biochemistry assessed at 0, 4, 8, and 12 weeks. Blood gas analysis was not performed in the control group.

The study design is illustrated in Figure 1. A total of 530 patients on maintenance hemodialysis were treated in our clinic during June 2012, including 32 with hyperphosphatemia who were taking oral sevelamer hydrochloride. Of these, 28 provided consent to switch from sevelamer hydrochloride to BXL (1:1 dose) from July to October 2012 (switch group). The control group consisted of 84 stable hemodialysis patients not taking sevelamer hydrochloride, randomly selected by random number tables using patient identification number. Patients were included if they (1) were aged 20 to 80 years; (2) were stable on hemodialysis sessions started at least 1 year before study entry; (3) provided written informed consent to participate; (4) had not changed their regimen of phosphate-lowering drugs, cinacalcet hydrochloride (if used), and other medications that could affect serum phosphorus levels for at least 28 days before study entry; (5) had not changed their dialysis regimens for at least 28 days before study entry; (6) had not changed other factors, including dietary therapy and concomitant drugs, during the study period; and (7) were in good general health, with an Eastern Cooperative Oncology Group Performance Status (ECOG-PS) grade of 0 or 1 [10].

Patients were excluded if they (1) had a history of gastrointestinal surgery (excluding polypectomy), dysphagia, ileus, gastrointestinal bleeding, severe persistent constipation or diarrhea, or had received parathyroid intervention within 3 months of study entry; (2) showed unstable control of serum phosphorus and calcium levels; or (3) were in poor general health, or had a major concomitant malignant disease or other medical condition likely to result in death within 6 months of study entry.

### Assessments

The primary endpoint was improvement of gastrointestinal symptoms. Secondary endpoints included improvements in metabolic acidosis, changes in blood biochemistry, and safety.

Gastrointestinal symptoms and form of stool were assessed at baseline (0) and 4, 8, and 12 weeks after switching in the switch group, and at 0 and 12 weeks in the control group. Hemoglobin, serum albumin, phosphorus, and calcium levels and metabolic acidosis were measured at 0, 4, 8, and 12 weeks in both groups. Gastrointestinal symptoms were assessed using the Izumo scale [11] and stool form was evaluated using the Bristol stool scale [12,13]. The Izumo scale is a self-reported questionnaire designed to determine the quality of life of patients with upper and/or lower abdominal symptoms. The questionnaire consists of 15 questions in five domains: reflux, stomach ache, epigastric fullness, constipation, and diarrhea. Each question is rated on a 6-point scale, from 0 to 5, with higher values indicating more severe symptoms. The scores of questions within a domain can be added to determine domain-specific scores.

The Bristol stool scale is designed to classify the form of human feces into seven categories: separate hard lumps, like nuts, which are hard to pass (Type 1); sausage-shaped, but lumpy (Type 2); like a sausage but with cracks on its surface (Type 3); like a sausage or snake, smooth and soft (Type 4); soft blobs with clear-cut edges, easily passed (Type 5); fluffy pieces with ragged edges, and mushy (Type 6); and watery, no solid pieces (Type 7).

Adverse events were evaluated according to the Common Terminology Criteria for Adverse Events (CTCAE), version 4.0 [14].

Satisfaction with BXL compared with sevelamer 12 weeks after the switch was assessed in the switch group using a 5-level rating questionnaire.

### Statistical analysis

Symptom scores were compared before and 3 months after switching in the switch group using paired *t* tests, and between the switch and control groups using Mann–Whitney *U* tests. Changes of metabolic acidosis and blood biochemistry parameters were compared before and after switching in the switch group using paired *t* tests. All statistical analyses were performed using GraphPad Prism version 5.03 (GraphPad software, Inc. La Jolla, CA, USA) with a P-value <0.05 considered statistically significant.

## Results

### Patient background

The background of the included patients is shown in Table 1. All were receiving hemodialysis. Overall mean age was 60.2 ± 10.8 years, 57 ± 12 years in the switch group and 61 ± 10 years in the control group, and overall mean duration of dialysis was 11.6 ± 6.5 years. Most patients (83%) had used other anti-phosphorus agents, including calcium-containing phosphate binders and lanthanum carbonate (Table 1). There were no significant between group differences in patient demographic and clinical characteristics, except for administration of potassium absorbent (*P = 0.047*), and components of other anti-phosphorus agents (*P = 0.001*). The mean dosage of sevelamer hydrochloride before the switch was 2054 ± 780 mg/day.

**Table 1:** Demographic profile of the subjects

	**All**	**Switch group**	**Ctrl group**	***P value***
n	112	28	84	
Age (Y)	60.2 ± 10.8	57.3 ± 12.0	61.2 ± 10.3	*0.134*
Gender (M/F)	55/57	14/14	41/43	*0.913*
Dialysis vintage (Y)	11.6 ± 6.5	12.1 ± 6.4	11.4 ± 6.6	*0.604*
Use of Psychotropic agents	39(35%)	12(43%)	27(32%)	*0.594*
Use of vitamin D analogues	46(41%)	14(50%)	32(38%)	*0.267*
Use of other phosphorus agents	93(83%)	24(86%)	69(82%)	*0.660*
Precipitated calcium carbonate	58(51%)	21(75%)	36(43%)	*0.001*
Lanthanum carbonate hydrate	12(11%)	3(11%)	9(11%)	
Precipitated calcium carbonate plus Lanthanum carbonate hydrate	24(21%)	0(0%)	24(28%)	
No agents	19(17%)	4(14%)	15(18%)	
Use of Potassium absorbent	47(42%)	17(61%)	30(36%)	*0.047*
Use of laxative	31(28%)	5(18%)	26(31%)	*0.179*
Cardiovascular disease	36(32%)	9(32%)	27(32%)	*1.000*
Diabetes mellitus	28(25%)	8(29%)	20(24%)	*0.614*

### Izumo scale and bristol stool scale

Gastrointestinal symptom analysis was performed after excluding the four patients who stopped taking BXL soon after switching. All symptom scores are summarized in Table 2. Total gastrointestinal symptom scores in the switch group 3 months after switching were similar to those in the control group. However, when we compared gastrointestinal symptom scores in the switch group before and 3 months after switching, we observed significant improvements.

**Table 2:** Changes in gastrointestinal symptoms, Bristol stool scale, and metabolic acidosis in patients switched from sevelamer hydrochloride to bixalomer and in control patients

	**Switch group, n = 24**			**Ctrl group, n = 84**			**Swich *vs. *Ctrl**	**Swich *vs. *Ctrl**
	**Before**	**3 months**	P value*	**Before**	**3 months**	P value*	P value** (Before)	P value** (3 months)
[Questionnaires]								
Reflux	2.0 ± 2.9	1.3 ± 2.4	*0.044*	1.3 ± 2.5	0.9 ± 1.8	*0.117*	*0.067*	*0.193*
Stomach ache	1.1 ± 1.6	1.1 ± 2.0	*1.000*	0.8 ± 2.0	0.6 ± 2.0	*0.422*	*0.057*	*0.122*
Epigastric fullness	3.0 ± 3.2	1.9 ± 3.2	*0.069*	1.5 ± 2.1	1.3 ± 2.4	*0.417*	*0.028*	*0.554*
Constipation	3.0 ± 2.9	1.5 ± 2.0	*0.004*	2.1 ± 2.4	1.5 ± 2.0	*0.073*	*0.143*	*0.855*
Diarrhea	2.2 ± 2.4	1.7 ± 2.1	*0.274*	2.1 ± 2.3	1.4 ± 2.2	*0.042*	*0.685*	*0.414*
Total score	11.3 ± 10.5	7.5 ± 10.1	*0.011*	7.9 ± 9.4	5.7 ± 8.2	*0.117*	*0.062*	*0.814*
Bristol scale	4.1 ± 1.0	4.0 ± 1.0	*0.704*	4.4 ± 1.1	4.1 ± 1.1	*0.083*	*0.209*	*0.594*
[Blood gas analysis]								
pH	7.36 ± 0.04	7.39 ± 0.04	*0.004*					
HCO3-	19.7 ± 2.3	21.3 ± 3.2		*0.028*					
BE (vt)	−5.0 ± 2.5	−3.3 ± 3.1		*0.012*					
Anion Gap	19.6 ± 3.3	15.8 ± 3.1		*0.000*					

*, paired *t* test, **, Mann–Whitney *U* test.

**Figure 2:**
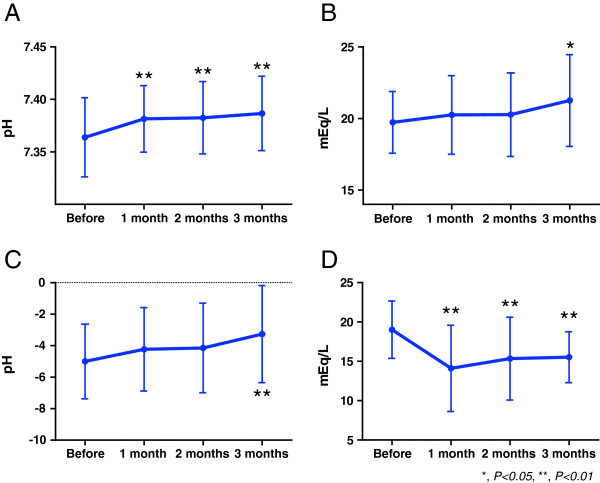
**Changes of metabolic acidosis.** All parameters of metabolic acidosis (**A**: pH, **B**: HCO_3_^-^, **C**: base excess, **D**: anion gap) improved significantly in the switch group. (*, *P < 0.05,* **, *P < 0.01, paired t tests, compared with before switching).*

Symptoms of epigastric fullness were significantly worse (*P = 0.028*) in the switch group before the switch than in the control group, but other symptoms did not differ significantly in the two groups. Twelve weeks after the switch, symptoms of reflux, epigastric fullness, and constipation had improved significantly in the switch group, resulting in no significant between-group difference in epigastric fullness. Other factors, including stomach ache, diarrhea, and the Bristol stool scale, did not differ significantly in the two groups (Table 2). Blood gas analysis showed that metabolic acidosis, including pH (Figure 2A), HCO_3_- concentrations (Figure 2B), base excess (Figure 2C), and anion gap (Figure 2D), were significantly improved in the switch group.

### Blood biochemical analysis

**Figure 3:**
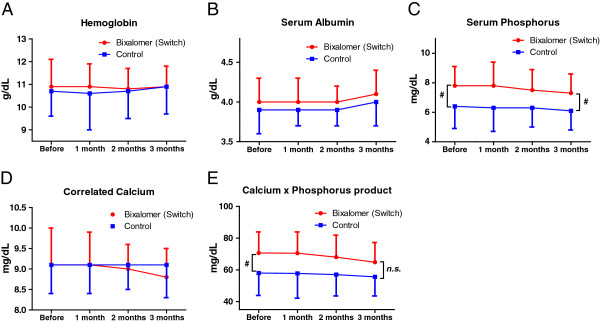
**Changes of blood biochemistry parameters.** Changes of blood biochemistry parameters in hemoglobin **(A)**, serum albumin **(B)**, serum phosphorus **(C)**, correlated calcium **(D)** and calcium × phosphorus product **(E)**. Serum phosphorus levels were significantly higher in the switch group before than after switching. No blood biochemical parameter changed significantly. (^*#*^*, P < 0.05, paired t tests, compared with the control group).*

Before the switch, serum phosphorus level (Figure 3C) and calcium × phosphorus product (Figure 3E) were significantly higher in the switch than in the control group. After the switch, the calcium × phosphorus product did not differ significantly in the two groups. There were no significant changes in hemoglobin (Figure 3A), serum albumin (Figure 3B), and calcium (Figure 3D) levels before and after the switch.

### Adverse events

Four patients in the switch group (14%) experienced CTCAE grade 1 adverse events, all of which improved immediately after stopping BXL treatment. The major adverse events were diarrhea (3.5%) and abdominal discomfort (3.5%). Details of adverse events are shown in Table 3.

**Table 3:** Adverse events and grade after switching

**Discontinuations patients**	
n	4 (14%)
Age (Y)	58 ± 11
Gender (M/F)	2 / 2
Dialysis vintage (Y)	17 ± 20
Administration periods (Day)	19 ± 20 (range: 1 – 45)
**Reasons of discontinuations**	**No. of cases (%)**
Diarrhea (Grade 1)	2 (3.5%)
Abdominal discomfort (Grade 1)	2 (3.5%)
Constipation (Grade 1)	1 (1.8%)
Nausea (Grade 1)	1 (1.8%)

### Satisfaction surveys

**Figure 4:**
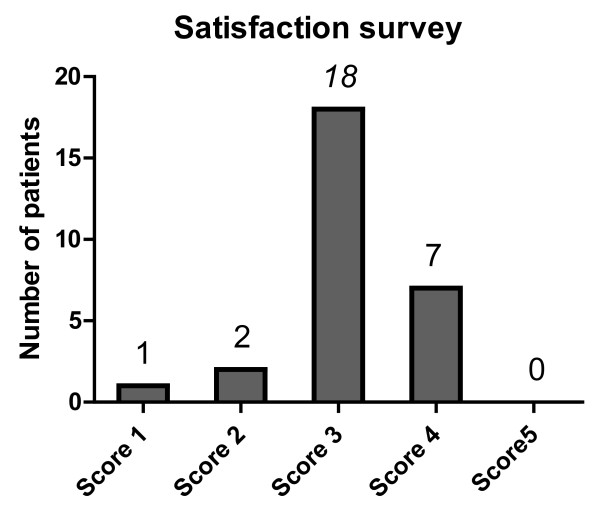
**Satisfaction survey for bixalomer.** Satisfaction with BXL was assessed by a 5-level rating, including “very dissatisfied (score 1)”, “somewhat dissatisfied (score 2)”, “neither satisfied nor dissatisfied (score 3)”, “somewhat satisfied (score 4)”, and “very satisfied (score 5)”. The most frequent answer after switching (64%) was “neither satisfied nor dissatisfied”, with only seven patients (25%) reporting being “somewhat satisfied” after the switch. The mean score was 3.1 ± 0.7.

Satisfaction with BXL was assessed by a 5-level rating, including “very dissatisfied”, “somewhat dissatisfied”, “neither satisfied nor dissatisfied”, “somewhat satisfied”, and “very satisfied” in all 28 switched patients. The mean satisfaction score was 3.1 ± 0.7, with most patients (64%) reporting that they were “neither satisfied nor dissatisfied” after the switch (Figure 4).

## Discussion

We have prospectively investigated the effectiveness and safety of a 1:1 dose switch from sevelamer hydrochloride to BXL in stable hemodialysis patients. We observed no significant difference in total gastrointestinal symptom score 3 months after switching in the switch compared with the control group. However, total gastrointestinal symptom scores in the switch group were improved 3 months after switching compared with before switching. We found that switching resulted in significant improvements in the Izumo scale, including reflux, epigastric fullness, constipation, and total score, suggesting that BXL may be better tolerated than sevelamer hydrochloride and may be an option for hemodialysis patients intolerant to sevelamer hydrochloride. To our knowledge, this is the first report showing the effects of switching from sevelamer hydrochloride to BXL.

A recent phase III trial comparing gastrointestinal symptoms in patients treated with BXL and sevelamer hydrochloride found that a significantly lower percentage of BXL patients experienced gastrointestinal symptoms [29% (16/55) vs. 47% (26/55), *P = 0.0497*] [15]. In addition, abdominal fullness was significantly less common in the BXL group (2% vs. 13%, *P = 0.023*). These results suggest that BXL may give rise to milder and fewer GI symptoms than sevelamer hydrochloride.

In contrast, a satisfaction survey showed that 64% of patients answered “neither satisfied nor dissatisfied” after the switch to BXL, with only seven (25%) reporting they were “somewhat satisfied”. These contradictory results may suggest that the reductions in gastrointestinal symptoms after switching may be limited. Because 86% of switched patients had previously used other phosphorus absorbing agents or other agents that induce gastrointestinal symptoms, any improvement in gastrointestinal symptoms did not have sufficient statistical power for clinical significance. Additional studies are needed to clarify this issue.

We observed that serum phosphorus concentrations did not change significantly after switching. Recent trials testing the efficacy of BXL for hyperphosphatemia found that it was non-inferior to sevelamer hydrochloride, suggesting that these two agents have similar effects on hyperphosphatemia [8,16].

As expected, improvements in parameters of metabolic acidosis were observed after switching. Similar outcomes were reported after switching hemodialysis patients from sevelamer hydrochloride to lanthanum carbonate, with significant increases in HCO_3_^-^ concentrations [17]. Additional studies are needed to address the long-term benefits of BXL in patients with metabolic acidosis.

The safety profiles of BXL were similar to those previously described [16]. BXL has shown long-term safety and efficacy in Japanese hemodialysis patients, with reasons for discontinuation including gastrointestinal disorders such as constipation and diarrhea. In this study, the major adverse events were CTCAE grade 1 diarrhea and abdominal discomfort. Although the grade of gastrointestinal adverse events was usually mild, these events occurred after switching from sevelamer hydrochloride, indicating the importance of monitoring hemodialysis patients, even those on BXL, for gastrointestinal adverse events.

This study had several limitations, including the small number of patients, the inclusion of patients within a single institute, and the non-randomized design. It is therefore difficult to control for selection bias and patient backgrounds. Randomized trials, involving larger numbers of patients from multiple institutions, are therefore necessary to assess the possible benefits of BXL.

## Conclusions

The results of this study demonstrated that gastrointestinal symptoms improved after switching to BXL, and that symptoms of epigastric fullness 3 months after switching were identical to those of the control group. BXL was well tolerated and may provide a new option in the treatment of hyperphosphatemia in patients undergoing hemodialysis.

## Abbreviations

DOPPS: Dialysis outcome and practice patterns study; BXL: Bixalomer; Ctrl: Control; CTCAE: Common terminology criteria for adverse events; ECOG-PS: Eastern cooperative oncology group performance status.

## Competing interests

The authors declare that they have no competing interests.

## Authors’ contributions

SH performed the statistical analysis, and drafted the manuscript. TK participated in drafting the manuscript. HM, TN, MO, NF, HI, JM, YK, TS, KF, YI, YH, and HS performed the clinical follow-up and contributed data to the manuscript. TF supervised the study. CO was responsible for the concept and design of the study, the interpretation of data, and critical revision of the manuscript. All authors read and approved the final manuscript.

## Pre-publication history

The pre-publication history for this paper can be accessed here:

http://www.biomedcentral.com/1471-2369/14/222/prepub
